# Data systems for the Linac coherent light source

**DOI:** 10.1186/s40679-016-0037-7

**Published:** 2017-01-14

**Authors:** J. Thayer, D. Damiani, C. Ford, M. Dubrovin, I. Gaponenko, C. P. O’Grady, W. Kroeger, J. Pines, T. J. Lane, A. Salnikov, D. Schneider, T. Tookey, M. Weaver, C. H. Yoon, A. Perazzo

**Affiliations:** SLAC National Accelerator Laboratory, 2575 Sand Hill Road, Menlo Park, CA 94025 USA

**Keywords:** Free-electron lasers, FELs, Serial femtosecond crystallography, Data acquisition systems, Data management systems, Computer programs

## Abstract

The data systems for X-ray free-electron laser (FEL) experiments at the Linac coherent light source (LCLS) are described. These systems are designed to acquire and to reliably transport shot-by-shot data at a peak throughput of 5 GB/s to the offline data storage where experimental data and the relevant metadata are archived and made available for user analysis. The analysis and monitoring implementation (AMI) and Photon Science ANAlysis (psana) software packages are described. Psana is open source and freely available.

## Background

Since the LCLS facility started operating in 2009, it has accumulated many petabytes of complex data for analysis, and the timely processing of this data has proven to be a challenge for the community. At LCLS, this has been made more difficult by the fact that experiments and experimenters change from week-to-week, and the fact that real-time feedback is often critical for making decisions on how to run an experiment. Furthermore, because of the intrinsic pulsed nature of the FEL source, experimental solutions must acknowledge that every shot is different and that a wide range of information needs to be recorded to interpret a single-shot event. The LCLS data systems must acquire all relevant shot-by-shot data at the 120 Hz repetition rate of the LCLS light source, provide user-friendly display and analysis of critical real-time information, write multiple GB/s to storage, and provide analysis software for the timely processing of this large and complex dataset. Each of the seven LCLS instruments [1, 2] offers unique capabilities to study many different areas of science using the unique FEL beam properties. Here, we describe the data acquisition (DAQ) and data analysis systems developed for LCLS and briefly describe a case study of the quasi-real-time nanocrystallography pipeline as an example of LCLS computing capabilities.

## Methods

### Data acquisition

The data acquisition system (DAQ) at LCLS is the set of hardware and software responsible for correctly and coherently transporting data from the instruments’ imaging detectors and diagnostic devices to a dedicated file system. The DAQ is used to configure, calibrate, and control both custom and commercial devices. Each instrument has its own independent DAQ system of hardware and software, allowing all instruments to be run simultaneously.

Within each instrument, data are acquired for all devices at the beam rate of 120 Hz, and UDP multicast from readout nodes over a dedicated 10 Gb network to several data cache nodes. The DAQ system performs an event build, the real-time assembly of the data from all devices into one object, called an event, tagged with the fiducial from the timing system and a UNIX timestamp. The data cache nodes subscribe to the UDP multicasts from the readout nodes, aggregate all device data associated with a single fiducial in an event, and append these event data to a file in eXtended tagged container (XTC) format [3]. The DAQ system is capable of reading out 5 GB/s per instrument, with the exceptions of the coherent X-ray imaging (CXI) instrument [4], which is capable of running two independent experiments simultaneously and whose infrastructure is capable of reading out 10 GB/s, and the matter in extreme conditions (MEC) instrument [5], which is limited to 1 GB/s due to its lower designed data rate.

By UDP multicasting the data for different events to different multicast groups, it is possible to scale the number of data cache nodes appropriately to accommodate large and small experiments. Multiple data files are written in parallel per run, one file per each of the six data cache node in a typical instrument. The multiple data files are recorded for a period of time called a run where an experiment has been taking data with a constant configuration. A run typically lasts between 10 and 40 min. Additionally, each of these files within a run is automatically split into chunks to prevent any one file from getting too large for the tape archiving system. The average file depends on the length of the run and for cyrstallography experiments it is typically 20 GB per file, six files per run.

Each hutch is equipped with dedicated monitoring nodes that also subscribe to the multicasts to receive a fixed fraction of all events where each event includes all the detector and diagnostic data recorded from one X-ray pulse. The monitoring nodes copy the data to shared memory where the data are promptly available for real-time analysis applications such as AMI or psana, as described below.

The data that arrive at the data cache nodes are stored in the SSDs while the transfer to the fast feedback (FFB) storage layer, which is initiated immediately when a run is started, completes. The transfer from data cache to FFB happens in near real time, or with a very small delay. The FFB layer can store 100–200 TB of data while awaiting transfer to permanent offline storage. Data can be accessed from disk, and custom analyses may be run on the fast feedback queues in each experimental hall. This method can provide quasi-real-time feedback within about 5 min of data acquisition. Access to the FFB storage layer is reserved to the running experiment.

**Fig. 1:**
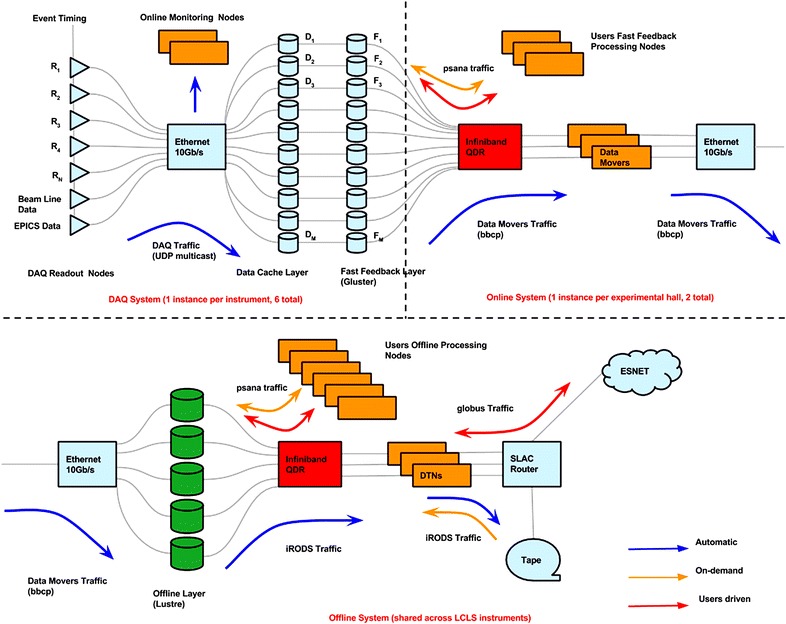
LCLS data flow. The *top half* of the figure represents the Online system which includes the DAQ and the Fast Feedback Layer. There is one Online system instance per instrument. The *bottom half* of the figure shows the Offline system which is shared across LCLS instruments. When the DAQ begins a new run for recording, the data management system ensures that the new files are registered in the file catalog and launches an automated process to immediately begin the transfer of data from the data cache nodes to the fast feedback (FFB) nodes as the raw data are being written

From the FFB, the data are automatically copied to the offline file system where files are made available to users for analysis and for exporting to users’ institutions outside SLAC via the POSIX-compliant Lustre file system [6]. The total data volume varies by experiment. Crystallography experiments typically generate a few hundred high-rate data bursts about 10 min in length over a 5-day period. An overview of the movement of data through the LCLS online and offline systems is shown in Fig. 1.

**Fig. 2:**
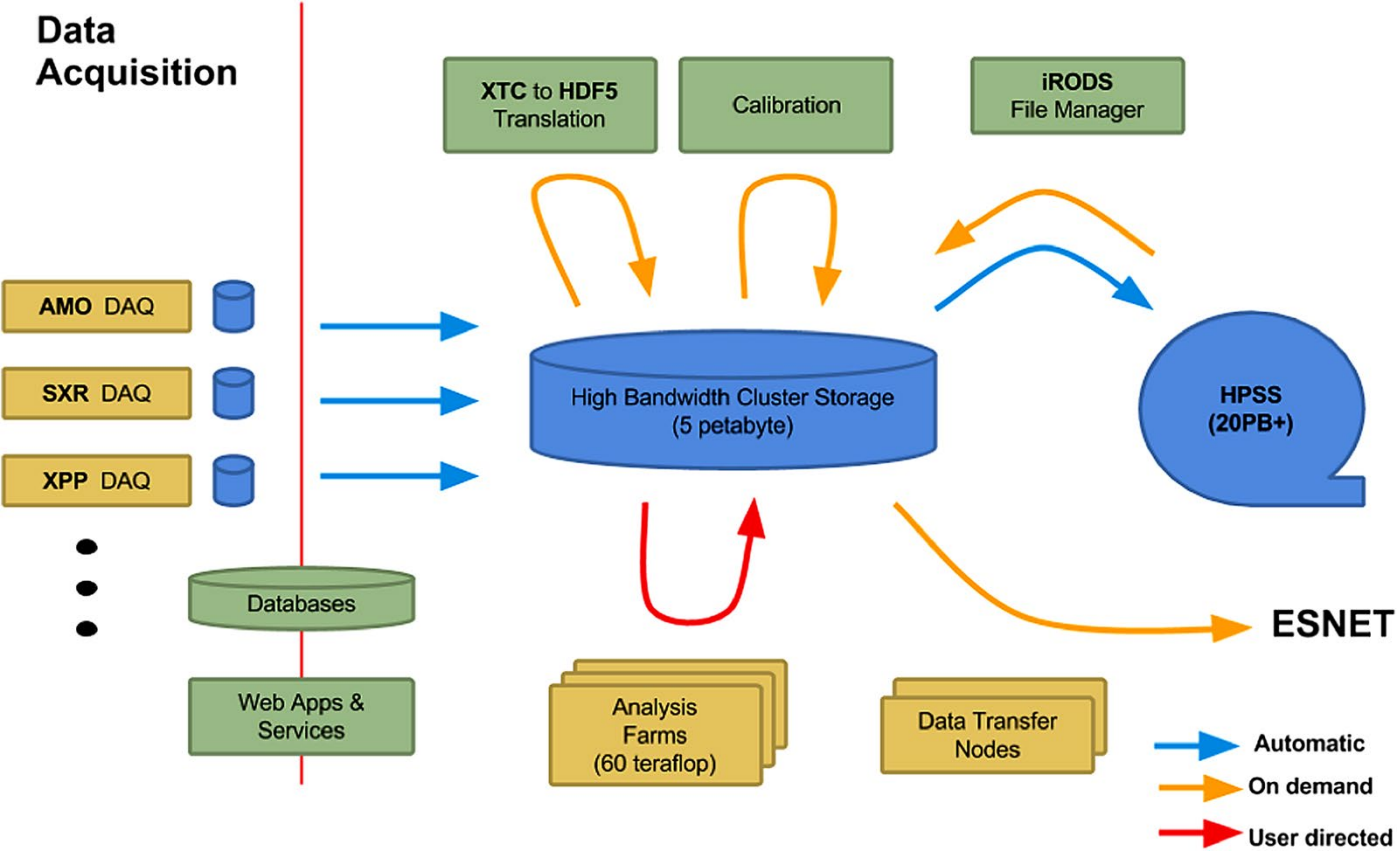
Logic diagram of the LCLS data management system. The *blue arrows* indicate data movement that is automatically handled by the DM system; the *red arrows* indicate traffic that is handled by the users; the *yellow arrows* show traffic that is handled by the DM system upon users’ request

In 2015, the LCLS Data Management system was expanded to include NERSC resources; after data are copied to tape at SLAC, the files are also copied to NERSC to create a second archive copy inAQ at NERSC. Simultaneous data migrations for all LCLS instruments are supported. The system maintains a central registry of experiments and provides a reliable mechanism for storing the data and metadata at the various storage layers of the LCLS computing infrastructure. Figure 2 shows a logical diagram of the LCLS data management system. Since 2014 [7], we have utilized the energy sciences network (ESNet) [8] to transfer data to NERSC, with sustained transfer rates of the order of 10 Gb/s. NERSC provides the significant computing resources of the Cori Phase I system [9]. Users may analyze the data at SLAC, at NERSC, or copy the data to their home institution and analyze it there.

The ability to make informed decisions in response to real-time feedback is critical during an LCLS experiment. It is essential for tuning the performance of the X-ray source, the detectors, and other beamline components. LCLS provides two software frameworks for displaying and analyzing critical real-time information: a graphical online monitoring tool called the analysis monitoring interface (AMI) and the software framework psana (Photon Science ANAlysis), a software package with user interfaces in both C++ and Python. All data generated by the DAQ can also be viewed and analyzed using this software.

## Results and discussion

### Data analysis using AMI

AMI runs alongside the data acquisition, is user-configurable, and requires no user coding or preparation to produce an analysis. AMI actually refers to a collection of software implemented in C++ and QT consisting of (1) a shared memory server, a generic application that receives datagrams from the DAQ private network via UDP, builds them into events, and pushes them into shared memory, (2) a custom application that receives these events from shared memory, performs analyses, and exports viewable data such as plots, and (3) online_ami, the QT-based GUI that runs on the control room consoles and serves as a network client to the ami server, receiving users’ analysis configurations and displaying resulting plots.

At the start of a run, the monitoring automatically learns which detectors are available in the data and makes their raw data available to the user with the click of a button. AMI is the default tool for real-time online analysis and feedback.

Shared memory analysis takes advantage of the fact that the LCLS data acquisition system uses UDP multicasts to simultaneously send data to the data cache nodes, that save data to disk, and to the monitoring nodes where data from the last 16–32 events are stored in a Unix shared memory buffer. The UDP multicasts are made pseudo-reliable by enabling hardware-based Ethernet pause frames to create backpressure in the network if buffers become full. If the monitoring code is too slow to analyze the full event rate, the oldest events are discarded, ensuring that the results are from the most recent data. Processes running on multiple cores can connect to the same shared memory server, which distributes different events to the different processes on the node and serializes client requests with datagram handling. The analysis results are then collected by a custom collection application and displayed to the operator by the online_ami client. AMI runs on an instrument’s monitoring nodes which typically contain over 40 CPU cores. There is one shared memory input per monitoring node, but multiple clients can coexist so that users may monitor the data on different consoles and using different criteria. The processing load is distributed across the monitoring nodes, but because each node receives complete events, it is capable of fully analyzing any given event.

Users primarily interact with the online_ami GUI and use it to display and analyze information on-the-fly. The GUI has a set of simple operations that can be cascaded to achieve a variety of monitoring measures. It can be used to perform many standard tasks such as displaying detector images and waveforms, displaying data as histograms, strip charts, scatter plots, etc., and performing averaging, filtering, and other generic manipulations of the data including region of interest selection, masking, projections, integration, contrast calculation, and hit finding. AMI can be used to view raw or corrected detector images and perform tasks such as background subtraction, detector correlations, and event filtering. For example, the analysis may require that only events in which the beam energy is above a certain threshold and a laser is present should be plotted. The plot can be further manipulated, overlayed on other plots, displayed as a table, or saved to a text file or an image. All of the scalar data such as the beam energy, beamline diode values, encoder readouts, and EPICS [10] data associated with the event are also available and can be combined in user-defined algebraic expressions. AMI supports single-event waveform plots and image projections which can be averaged, subtracted, and filtered. AMI has an algorithm for simple edge finding using a constant fraction discriminator. Displays of waveforms and images can be manipulated by adding cursors and doing cursor math or waveform shape matching. Users may also integrate their own code to perform even more sophisticated or device-specific processing, either by building a C++ module plug-in for AMI, or writing Python code to run in the psana framework. AMI algorithms are available from our Subversion repository, https://confluence.slac.stanford.edu/display/PCDS/Software+Repository. Instructions for code development are documented here: https://confluence.slac.stanford.edu/display/PCDS/AMI+Online+Monitoring.

**Fig. 3:**
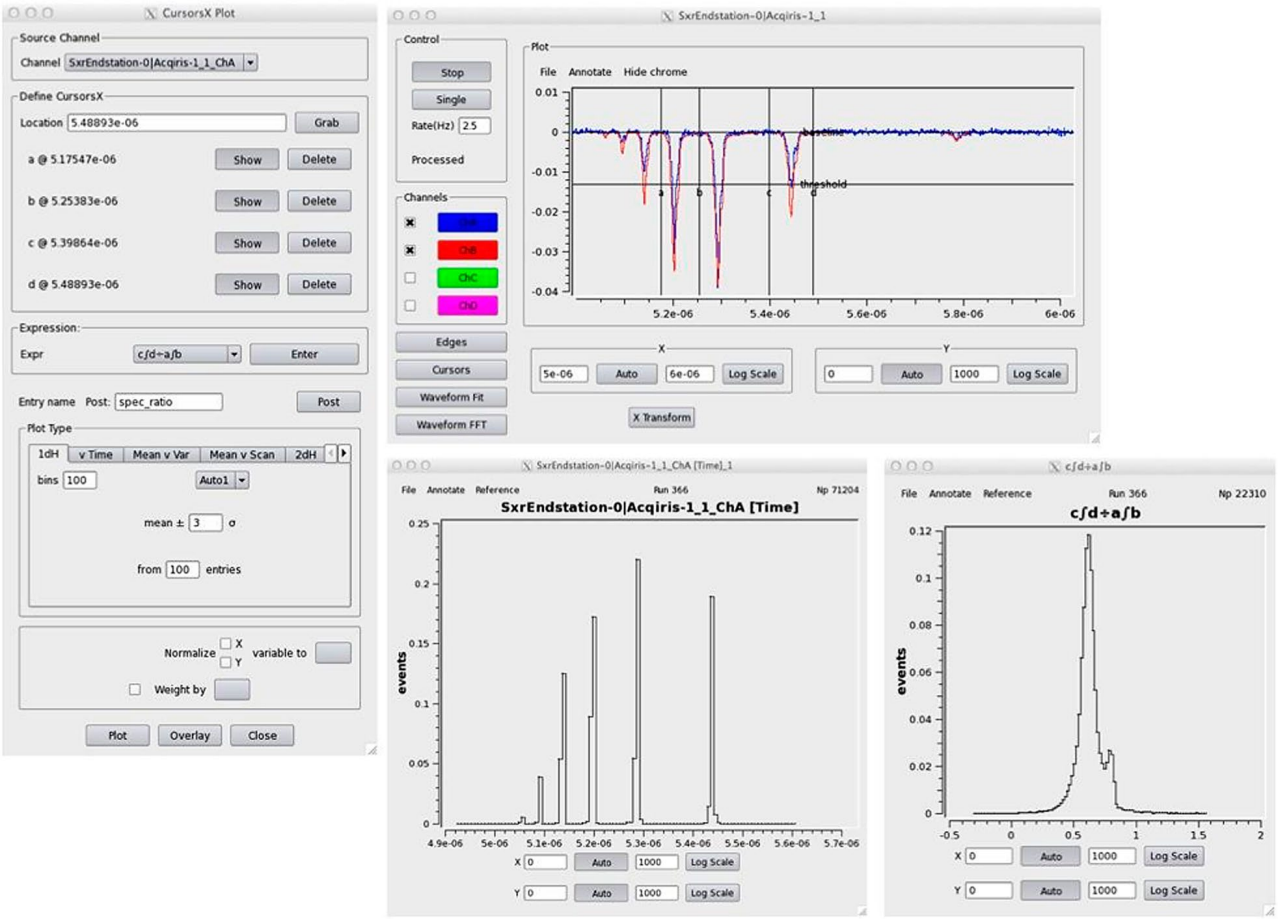
Example of event waveform plots and cursor math in AMI. The *top right image* shows the raw waveform in *blue* with the averaged waveform in *red* superimposed, and a baseline and threshold for the edge finding. The users have placed cursors on the image to select regions of interest. The *leftmost* window shows which channel is selected, the positions of the cursors on the plot, and the expression derived from the waveform. The plot in the *bottom right corner* is a 1D histogram expression derived from the waveform, histogramming the ratio of two areas selected by cursors

**Fig. 4:**
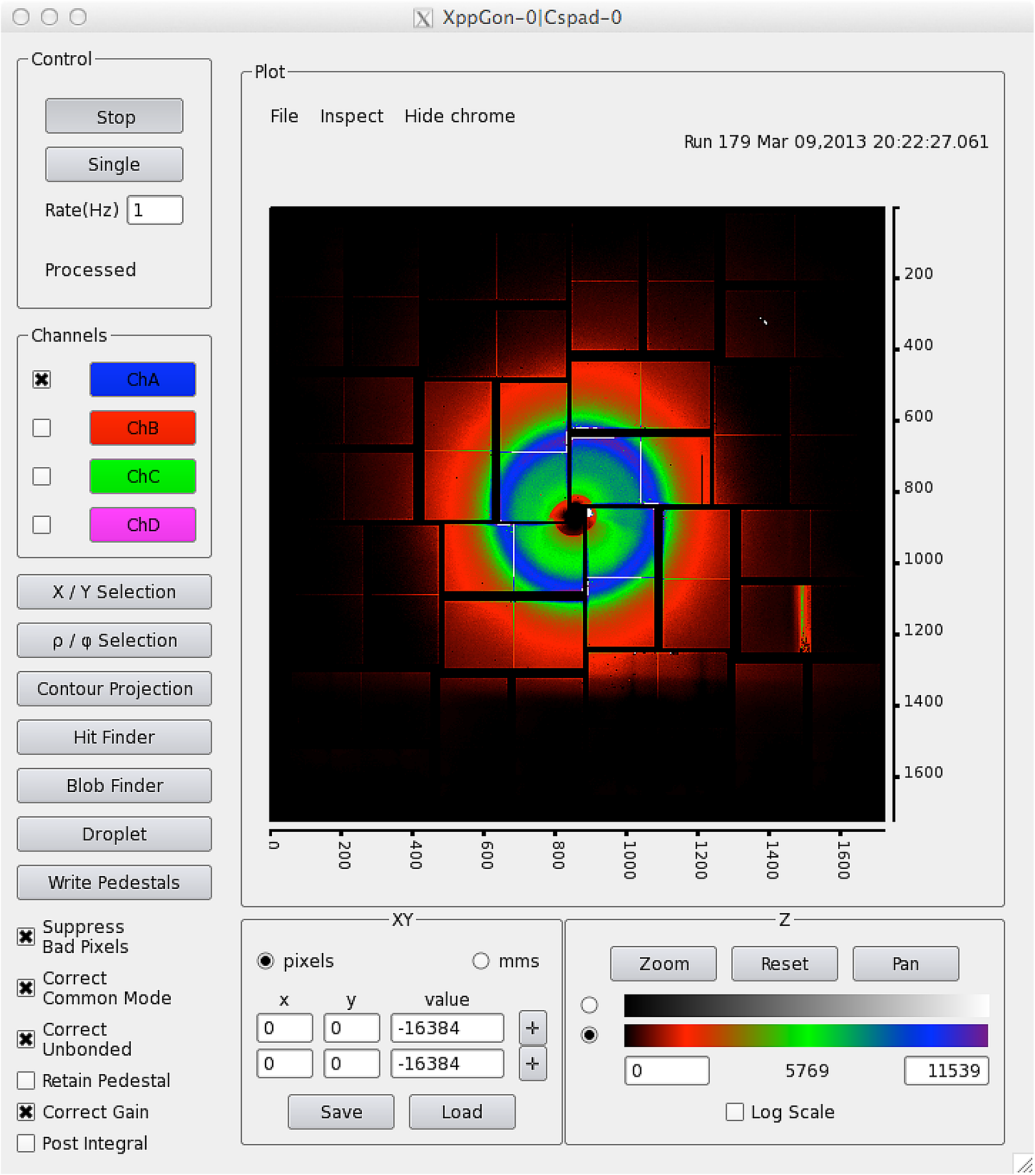
AMI screen capture of CSPad image. Screen capture showing CSPad [40, 41] as it appears during an experiment

AMI can be used both on live data from shared memory and offline data read from disk without any coding. Figures 3 and 4 show examples of AMI waveform analysis and image displays. AMI is a useful tool for generic online analysis and feedback, but psana is a more comprehensive analysis tool available to support more experiment-specific analyses.

### Data analysis using psana

The software framework psana handles importing the science data into memory (either staged from disk or streamed directly from the detectors), calibration, distributing events to multiple nodes/cores for parallel processing, and collecting the results and making them persistent. The psana framework is responsible for loading and initializing all user modules, loading one of the input modules to read data from XTC or HDF5 [11] files, calling appropriate methods of user modules based on the data being processed, providing access to data as a set of C++ classes and Python classes, and providing other services, such as histogramming, to the user modules.

The core portion of psana is written largely in C++, but psana supports both C++ and Python as user interfaces. Over time, it has become clear that Python is the preferred user interface for several reasons. First, it is possible to develop python analyses quickly, and short development times are a necessity given the frequent rate-of-change of LCLS experiments and the changing analysis requirements during an experiment. Second, C++ offers a steep learning curve for users. The observed trend at US light-source facilities and free-electron lasers around the world is to use Python and its associated tools.

In addition to providing data access, psana also provides simple python interfaces to complex algorithms. One commonly used example is the analysis code for the XTCAV detector [12] that is used to calculate lasing power as a function of time (on the femtosecond time scale) for each LCLS shot. Another example is the algorithm which computes the time separation between a pump laser and the LCLS shot [13]. Users are able to put together short python building blocks to quickly express the complexity of their experiment. Many of these building blocks are publicly available on the web, and so can be reused at any facility. We hope to include algorithms that are not LCLS-specific in globally available photon science-specific python packages which can be reused across labs. One such candidate is the publicly available scikit-beam project [14]. Psana and all its algorithms are open source and freely available from our Subversion repository. Instructions for code development and collaborative tools are documented here: https://confluence.slac.stanford.edu/display/PCDS/Software+Repository.

For performance, we support running psana in parallel using OpenMPI [15] through the python wrapper MPI4Py [16]. Several other photon science analysis packages [17] reuse the psana code when running at LCLS: OnDA [18], Hummingbird [19], cctbx.xfel, the Computation Crystallography Toolbox [20], the CrystFEL package [21], and Cheetah [22].

#### Interfaces

The data acquisition system is obligated to record all possible information to the data files, but the resulting complexity makes navigating the data difficult for the users. As a result, in addition to an interface that provides access to all data, we have found it useful to provide an additional simpler interface that exposes only information that most users typically access. We have also used this interface to capture commonality among detectors, e.g., all area detectors are transformed at a low level into NumPy arrays, either two-dimensional for a standard camera, or three-dimensional for multi-panel cameras. This is a powerful idea: metadata associated with a detector, such as pedestals, masks, per-pixel gains, can be given the same array shape as the real data, and then data corrections become efficient single-line NumPy operations like addition, multiplication, etc.

For performance, it is important that Python is able to call C++. For this, we have written Boost.Python (http://www.boost.org) converter methods for a few high-level classes that allow transfer of data between Python and C++ without copying large data. Memory management is done mostly in C++ using reference counts. We also use Boost.Python wrappers to call C++ class methods from Python. This allows for event analysis in a combination of C++ and Python, although the large majority of users only see the simpler Python interface.

#### Random access and parallelization with psana

MPI is a world standard for scientific parallelization across multiple nodes, with each node having many CPU cores. For most LCLS analyses, events can be analyzed in parallel, and I/O is a common bottleneck, which can be addressed using multiple cores/nodes. Most LCLS analyses parallelize trivially, with different cores processing different events. The psana MPI process running on a given core/node needs a way to jump to the events it will process—that is it needs random access to the large data rather than having to read through all the data. To achieve this, the data acquisition system writes additional small files called small-data XTC files where each piece of large data (e.g., a camera) is replaced with a file-offset into the full-data files. We maintain the same XTC format as the full data in these small-data files so that the same tools can be used to read it. When running with MPI, each core quickly reads these small-data files and then jumps to the appropriate big data for events that it should analyze by passing the big data file-offset to the fseek subroutine. Currently, the threshold for deciding which data is large or small defaults to 1 kB, but it can be overridden on the command line of the data acquisition software that records the data.

Further performance gains can be obtained from this small-data approach. For example, when processing an event, one can query beam quality (contained in the small-data files) and if the X-ray shot power was too low avoid spending the time to read the large data for that event. Psana has been structured so this conditional fetching can be done with a simple python “if” statement.

Psana also implements a user interface, based on random access, which accepts an event identifier and immediately returns the appropriate event. This identifier is the Unix seconds/nanoseconds timestamp plus a 17-bit 360 Hz “fiducial” counter as described previously.

#### Real-time analysis with psana

Prompt analysis of the data is critical for LCLS experiments, because such information is required for important decisions, e.g., beam tuning, moving detectors/samples, and evaluating whether or not sufficient statistics have been accumulated. It is possible to run psana data analysis in real time in two different modes, a shared memory interface, which receives DAQ network-multicast data, or a live-file mode where the data are read from the FFB storage layer:

1. In the shared memory mode, psana reads events from a shared memory buffer on the monitoring node and uses MPI to launch processes on the different nodes for full 120 Hz analysis.
2. In the FFB mode, the data acquisition small-data XTC files can be analyzed with MPI while the data are being written. If the software catches up to the end of the live file in this mode without seeing an end-run message, it will briefly sleep and try to read new data. If no new data appear within a timeout period, the software assumes no more events will appear and behaves as if the run had ended normally, albeit with a warning message.

The two online analysis approaches are complementary: FFB allows the user to analyze all events, at the risk of falling behind; shared memory has only a small buffer of events, meaning that the displayed data are always up to date, but there is no guarantee that all events will be seen by the analysis software, i.e., if the software is too slow, events will be dropped. Further, psana allows the user to run the same analysis code in online against the shared memory, quasi-real-time against the files on the FFB, and offline against data stored on disk.

#### Real-time visualization with psana

In addition to the standard matplotlib [38] methods for visualization in Python, we have used PyQtGraph to support real-time visualization because it has excellent interactive manipulation tools for plots together with fast graphics performance. The Python interface of the ZeroMQ (ZMQ) package [23] is used to transport data between the analysis code and the display, which may be on a remote machine. We use the publish/subscribe mechanism of ZMQ so that many real-time copies of plots may be displayed on different computers. To open a display, the subscriber uses a one-line command, which specifies the publisher’s hostname and port number, as well as a list of plot names.

Users can also create a multiplot which guarantees that all plots within the multiplot display coherent information, e.g., from the same LCLS events. In parallel jobs, typically one core is chosen to gather the results from the other cores via MPI and then publish the plots.

#### Build/release system

We use the SCons tool [24] to build all core Python/C++ packages of psana. The RHEL 5/6/7 operating systems are currently supported. All psana core and external packages are distributed using a modified form of APT [25] that supports relocatable RPM files. The repositories are made world-readable via http, so any user can download/run the APT code from the SLAC servers and quite easily install all psana binaries on a supported operating system. With the recent emphasis on Python-based analysis, we are considering a more Python-oriented release system, such as Anaconda [26], which would allow easier inclusion of Python external packages.

### Detector calibration

LCLS supports calibrations of several area detectors, many of which have multiple panels. These calibrations include pedestal subtraction, bad-pixel determination, and common-mode noise removal, where noise varies coherently in several channels of a detector in one event. All corrections are stored in a run-dependent manner, e.g., pedestal values, common-mode noise parameters. The calibration data are stored in a hierarchical directory structure: with an experiment containing several detectors, each of which has several parameter types and run-associated data files. We considered storage in a database, but felt that a simple directory structure would allow for easier portability of analysis to remote institutions. Most of the constants are stored in text files, but we anticipate storing future constants in hierarchical HDF5 files. The same file-based constants are used by both offline and online analysis, including the AMI tool.

Command line and GUI tools are provided to compute pedestals, noise values, and bad-pixel lists. The graphical interface allows users to take appropriate multi-panel unassembled detector data, e.g., powder-pattern diffraction-ring data and graphically adjust the positions/rotations of the panels to create geometry constants. Optical measurements with a microscope and sophisticated crystallographic techniques [27] are used to more precisely determine geometry. The tools are used to deploy calibration constants that are valid for user-specified run ranges.

**Fig. 5:**
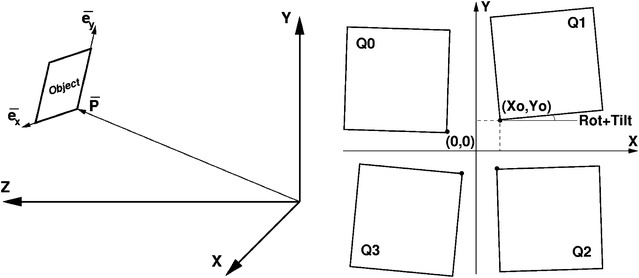
Hierarchical geometry description used by psana. *Left* one level in the hierarchical geometry description used by psana showing a child object in the parent coordinate frame. *Right* several panels of a multi-panel detector showing rotations and offsets. Although not shown in the diagram, the hierarchical geometry description allows these to be out-of-plane

Geometry for multi-panel detectors is defined using a multi-level hierarchical approach as shown in Fig. 5; each component is positioned with parameters defining its rotation and translation in the parent frame. Multiple independent detectors can be placed in the correct position relative to each other using this approach. In many experiments, the origin is defined as the interaction point between the sample being studied and the laser shot.

### Data type and data format

The data acquisition system produces many data types, implemented as C++ classes, and often these data types change with time as improvements are made. These changes are handled by introducing a new type for each modification using a custom-built data definition language (DDL) that allows us to represent the various data types in a language-independent manner. These descriptions are then compiled into language-specific Python or C++ classes. The DDL files are shared in common with the data acquisition system software, which uses C++, to guarantee a consistent description of LCLS data types between online/offline Python/C++ code.

The LCLS data acquisition system saves data in XTC format which consists of a hierarchical set of small headers that encapsulate larger data, where each container is mapped to a C++ class using an enumerated type. In the case of a dropped packet or missing data contribution, the header metadata associated with the event is annotated appropriately. It is an append-only data format, and only supports little-endian machines.

All code for writing/reading XTC data is contained in a library called pdsdata which has minimal dependencies. All data needed for analysis, including low-rate monitoring data like temperatures/voltages, exist in the XTC files. Because there are multiple files per run, easy user analysis requires a software framework like psana to manage the data reading. Psana presents the events from the multiple files to the user in time order, as well as doing offline event building when required. While the DAQ system performs real-time assembly of data from different detectors belonging to the same FEL shot into an event such that each XTC file is typically a sequence of complete events, there are also detectors that are shared across multiple data acquisition systems, although not simultaneously, and their data files are recorded separately and not included in the online event building process. To make these detectors easily available to users’ analysis code, psana additionally performs an offline event build that associates these data with the data acquisition data using the same timestamp, but at the time when the data are being read for analysis.

Because some users prefer HDF5 for offline analysis, the system provides a user-selectable translation service that can be configured from the LCLS web portal application to run automatically on the FFB queues and translate the raw XTC data to HDF5 as the data are being taken. The service produces raw or calibrated data organized into datasets based on each device rather than events. In addition, the data are self-describing with no software infrastructure required for analysis. The HDF5 data file has hierarchical organization consisting of the *groups* and *dataset*. Groups can contain other groups and datasets; datasets contain complex multi-dimensional data. This allows easy navigation from the “top” of the file to any object in that file, for example, /groupA/groupB/dataset1.

Users can take the data files off-site and analyze them in MATLAB, Python, or any other system that reads HDF5. Users can also customize the output of the translator by providing a configuration file to specify which data types should be translated or by including code that generates n-dimensional arrays which will automatically be included by the translator in the output.

While users do not need a software framework to work with LCLS HDF5, they all need to write the same code to correlate data from different datasets. That is, they need to match timestamps from the different datasets that the translator writes. This is essentially the event building process that psana must do with certain detectors. It is anticipated that as part of the LCLS-II upgrade the data acquisition system will write HDF5 files directly, given a couple of new critical features in the HDF5 1.10.x series, namely the ability read while writing and the ability to write to multiple files in parallel and aggregate them into one virtual dataset.

### Analysis computing resources

LCLS has accumulated 11 PB of data since start-up in 2009, and 24% of these data are currently available on disk. Frequently, the data acquisition rate is more than 1 GB/s. For analysis, we provide 80 nodes each with 2 Xeon X5675 processors and 24 GB of memory. These nodes use a 40 Gb/s infiniband connection [28] to access data on Lustre file-systems [6] providing a total of 3.7 PB of offline storage. Additionally, running experiments have special priority access to 2 additional farms of 20 nodes, each with 2 Xeon E5-2640 processors and 128 GB of memory. These nodes are used for prompt data analysis against the FFB layer and are reserved for the running experiment using the standard SLAC batch system. These nodes can also be used for general lower-priority jobs, which are automatically suspended when the higher-priority jobs of the running experiment are submitted.

### Case study: serial femtosecond crystallography

About one-third of beam time allocations at LCLS are currently awarded to serial femtosecond crystallography (SFX) experiments. With LCLS, it is possible to probe the sub-picosecond time domain, e.g., by triggering chemical changes with an optical pump/X-ray probe arrangement [29], or to observe sub-populations of conformational variation in the protein ensemble that are key to understanding enzyme mechanism and regulation [30].

The primary issue in XFEL crystallography processing pipelines is orchestrating movement of images through machine’s memory hierarchy as efficiently as possible while concurrently scheduling analysis tasks. This section describes the SFX pipeline based on cctbx.xfel [20], the computation crystallography toolbox, but other tools, like the CrystFEL package [21], are also available to the LCLS users.

Raw data from the X-ray sensors and from various diagnostic detectors are streamed at a sustained transfer rate near 10 Gb/s. With present data rates (120 Hz repetition rate and average image size of 4.5 MB), steady-state parallel analysis has been demonstrated, with the data being processed at the same rate they are acquired, by distributing the individual images to separate cores over multiple nodes [31]. Structural information is derived from the diffraction data collected from a stream of individual crystals. The Bragg spot intensities on each diffraction pattern are measured using the program cctbx.xfel. Four steps are executed in sequence: spotfinding (the identification of bright X-ray diffraction spots), indexing (the determination of the initial lattice model), refinement (parameter optimization for the lattice model), and integration (best-fit intensity modeling for individual Bragg spots). Simple parallelism is achieved by allocating each image to a different core. This level of parallelization is sufficient to keep up with current data rates with current analysis techniques, hence there is no present need for intra-image parallelism.

The top-level data reduction code from cctbx is called from within a psana script, which uses MPI to distribute the data. Concurrent processing is performed on approximately 1200 cores, corresponding to about 50 TFLOPs. This basic algorithm in the feature extraction pipeline for SFX image data from LCLS requires ~10 s/image single-threaded on a Xeon processor. Each of the four steps in the algorithm takes ~2.5 s to complete. The overall cycle time from data acquisition to reduced data is about 10 min.

An alternative SFX pipeline using psocake for spotfinding takes approximately 1.1 s/image to complete. Indexing and integration steps in CrystFEL take ~10 s/image; however, 95% of this time is spent reading an input hdf5 file containing the detector images and the spotfinding results suggesting huge gains can be achieved by bypassing the filesystem.

The current algorithms for SFX use the coarse approximation that each Bragg spot is located at a discrete mathematical point on an idealized lattice, with signal represented by summation of nearby pixel intensities. It has been shown that more accurate analysis is possible with protocols needing 100- to 1000-fold more CPU time [32].

#### Psocake

Since a typical LCLS experiment has millions of snapshots to choose from, it is critical to provide a means to quickly select images of interest and set regions of interest using masks. Included in psana is a graphical user interface called psocake [33] for viewing Area Detector images (CsPad, pnCCD, Opal, etc.) and that can be used to tune peak finding parameters and more closely examine the data. For example, one can mouse over a detector pixel display and identify its *x* and *y* pixel position and the ADU value. Regions of interest can be selected, masks can be drawn and applied, and events can be browsed using forward and back buttons. The user may save any event displayed as a NumPy array and can load and apply NumPy arrays to the image. For example, there is an option to launch an MPI job that saves a virtual powder pattern (mean, std, max) in a NumPy array. Users can click a button to optimize hit finding parameters, hit finding algorithms, and common-mode correction parameter for their experiment. Psocake and the algorithms are freely available from our Subversion repository: http://java.freehep.org/svn/repos/psdm/list/.

**Fig. 6:**
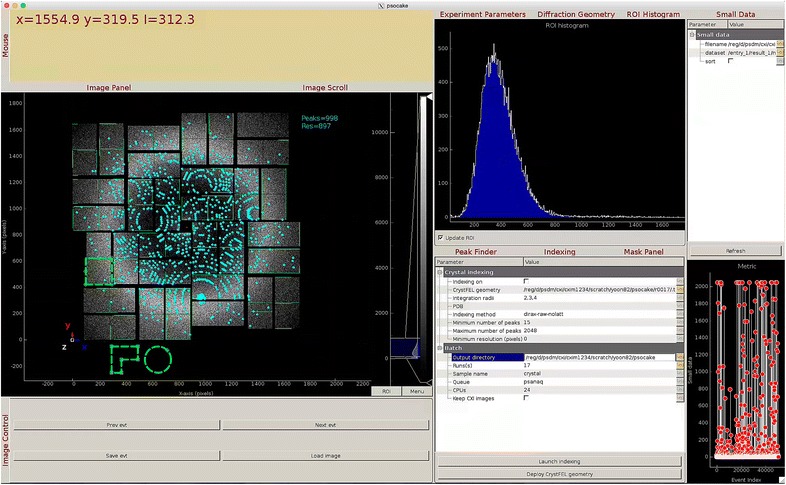
Screenshot of psocake tool. At *left* is the raw image with found peaks shown in cyan. At *right* is a histogram and information panel showing details about the peaks found in the selected region of interest

From within psocake, the user can tune hit finding parameters and launch peak finding jobs on multiple runs. The results of these jobs, the number of peaks found for each event, may be plotted (and refreshed) within psocake while the jobs are still running. By clicking on the plot, one can jump to the corresponding event and easily browse over the most interesting images based on the number of peaks. Psocake will also assist the user in doing crystal indexing using accurate detector geometry. Figure 6 shows an example of the psocake tool being used to inspect peaks found in an image.

### Architectural choices

The main difference between our system and other comparable systems, especially those found in high-energy physics (HEP) experiments, is the lack of a veto or trigger system. While a veto mechanism is part of the design, it was never deployed because of the following reasons:

- Many LCLS experiments have hit rates close to 100%, i.e., most pulses produce useful events. This is fundamentally different from most HEP experiments where the rate of a specific physics process is limited by the cross section of that process. This implies that the LCLS DAQ system had to be designed to handle the full machine rate.
- Experiments change on weekly basis: these changes are often profound enough that adapting the veto/trigger parameters and algorithms to each experiment would represent a huge effort.
- At the 120 Hz repetition rate of the source, and the average size and quantity of sensors, our current system can sustainably read out all data from all sensors at the full rate without the need for a mechanism to reduce the data on the fly.
- Finally, obtaining the buy-in and the collaboration of the various experimental groups in determining the right parameters and algorithms for selecting data on the fly proved very difficult.

Because of the cost of building and maintaining a large storage system, we encourage the users, through the retention policy, to keep only the useful data on disk. Data may be reduced in offline processing and selectively saved to disk, although a full copy of the raw data is still preserved on tape.

Another characteristic of the LCLS data system is the presence of multiple storage layers (data cache, fast feedback, and offline, as shown in Fig. 1). As discussed above, it is critical for the users to be able to perform prompt analysis on the data. While the separation between quasi-real-time and offline processing resources can be handled relatively well via the enforcement of high- and low-priority processing queues, the storage aspect was best handled by the introduction of dedicated resources for the running experiment. The separation between data cache and fast feedback is dictated by the need to separate the DAQ writes from the user activities. We believe this separation will not be necessary in the future with the adoption of flash-based storage technologies that handle much better concurrent access from different sources.

## Conclusions

The adoption of a language standard such as Python would allow scientists to move across facilities and reuse familiar low-level, publicly available tools. It is typically difficult to port large high-level frameworks to different facilities: it is easier to make low-level standard building blocks reusable. Examples of low-level, publicly available Python tools that we currently reuse that are useful for photon science include h5py [34], PyQtGraph [35], SciPy [36], NumPy [37], matplotlib [38], and MPI4py [39].

In order to enable faster feedback for experiments, we hope to explore graphical options, similar to the techniques used in the current C++-based LCLS online AMI GUI package, but implemented in Python for increased flexibility and decreased development time.

The upcoming LCLS-II upgrade with its 1 MHz repetition rate and potentially very high throughput (>100 GB/s) will necessitate an upgrade of the data acquisition and data processing capabilities. In general terms, the main challenge for the offline computing infrastructure will be developing high-throughput, high-density, peta-scale storage systems that allow concurrent access from thousands of jobs.

In the high-throughput regime, unlike in LCLS-I, it will be necessary to reduce the data prior to writing it to persistent storage. We are investigating the possibility of a data reduction mechanism through lossy compression to extract the key features from the data thus reducing the overall throughput. Note that a veto system alone will not be enough to reduce the data, since, like in LCLS-I, many experiments are expected to have close to 100% hit rate. Also, to participate in the veto system, sensors would need to provide a signal to the timing system which requires a custom interface that, although possible for custom-built sensors, would potentially make impractical the adoption of detectors developed elsewhere.

**Fig. 7:**
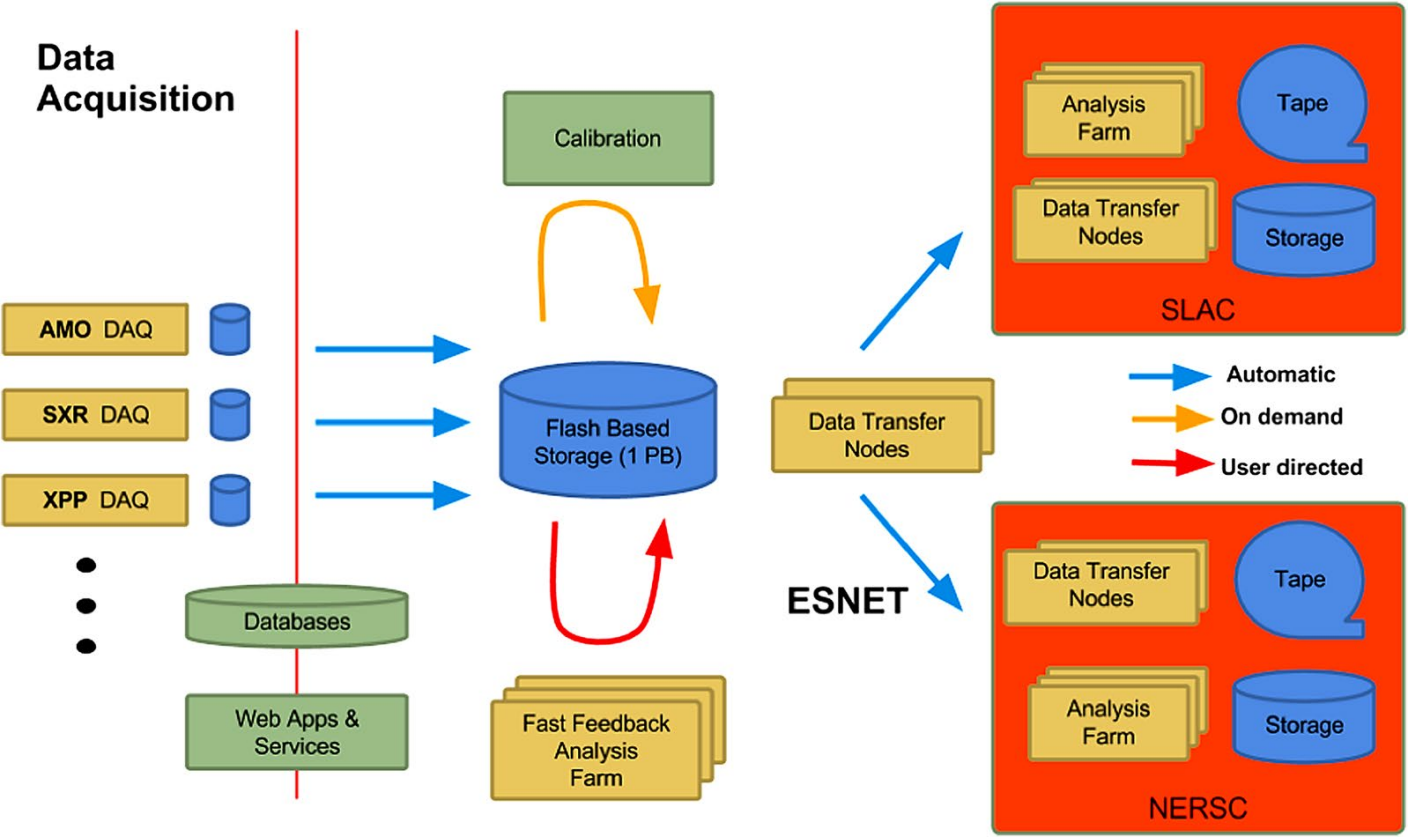
Evolution of the LCLS data systems architecture. The data management system will transparently integrate external supercomputers from facilities like NERSC

We plan to leverage DOE supercomputer facilities by offloading experiments with the highest processing needs (>10 PFLOPS) to NERSC. Expanding the existing collaboration with NERSC will avoid the need to scale the high-performance computing (HPC) capabilities at SLAC to the highest demand experiments, 10–1000 PFLOPS scale, while maintaining critical capabilities at SLAC. Figure 7 shows how the LCLS data systems architecture will evolve to integrate external computing facilities.

While we believe that well-scheduled intense bursts of computing power, well-coordinated over powerful networks, significantly expand the possibilities of fast feedback analysis for FELs, we face key challenges to our ability to run the LCLS analysis on NERSC supercomputers:

- The throughput of the required WLAN connection will be at the technological limits of what will be available in the LCLS-II timescale.
- Methods for data reduction or compression must be included. We anticipate that some analysis stages, especially data reduction stages that are not compute intensive, may be best placed close to the detectors.
- The extreme burstiness of the data creates new scheduling and data management challenges not common in supercomputers.
- Because one of the key goals is fast feedback, interfaces and components for in situ visualization of results will be key. For debugging, it will be necessary to be able to attach visualization and feedback components to any stage of the pipeline.
- The psana code will need to scale from the current hundreds of cores to hundreds of thousands.

## Abbreviations

ADU: analogue digital unit
AMI: analysis and monitoring implementation
APT: advanced packaging tool
CPU: central processing unit
CXI: coherent X-ray imaging
DAQ: data acquisition
DDL: data definition language
EPICS: experimental physics and industrial control system
ESNet: energy sciences network
FEL: free-electron laser
FFB: fast feedback
GB: gigabyte
Gb: gigabit
GUI: graphical user interface
HDF: hierarchical data format
HEP: high-energy physics
HPC: high-performance computing
LCLS: Linac coherent light source
MEC: matter in extreme conditions
MPI: message passing interface
NERSC: National Energy Research Scientific Computing Center
PSANA: photon science analysis software
PFLOPS: petaflops
RPM: RPM Package Manager
SFX: serial femtosecond crystallography
SSD: solid-state drive
TB: terabyte
UDP: user datagram protocol
XTC: eXtended tagged container
XTCAV: X-band radio-frequency transverse cavity
ZMQ: ZeroMQ, a high-performance asynchronous messaging library
